# Limited connectivity and a phylogeographic break characterize populations of the pink anemonefish, *Amphiprion perideraion*, in the Indo-Malay Archipelago: inferences from a mitochondrial and microsatellite loci

**DOI:** 10.1002/ece3.1455

**Published:** 2015-03-25

**Authors:** Tina A Dohna, Janne Timm, Lemia Hamid, Marc Kochzius

**Affiliations:** 1 Biotechnology and Molecular Genetics, UFT, University of BremenBremen, 28359, Germany; 2 Marine Biology, Vrije Universiteit BrusselBrussel, Belgium

**Keywords:** Coral Triangle, Indo-Pacific barrier, marine conservation, mito-nuclear

## Abstract

To enhance the understanding of larval dispersal in marine organisms, species with a sedentary adult stage and a pelagic larval phase of known duration constitute ideal candidates, because inferences can be made about the role of larval dispersal in population connectivity. Members of the immensely diverse marine fauna of the Indo-Malay Archipelago are of particular importance in this respect, as biodiversity conservation is becoming a large concern in this region. In this study, the genetic population structure of the pink anemonefish, *Amphiprion perideraion*, is analyzed by applying 10 microsatellite loci as well as sequences of the mitochondrial control region to also allow for a direct comparison of marker-derived results. Both marker systems detected a strong overall genetic structure (Φ_ST_ = 0.096, *P* < 0.0001; mean *D*_est_ = 0.17; *F*_ST_ = 0.015, *P* < 0.0001) and best supported regional groupings (Φ_CT_ = 0.199 *P *<* *0.0001; *F*_CT_ = 0.018, *P *<* *0.001) that suggested a differentiation of the Java Sea population from the rest of the archipelago. Differentiation of a New Guinea group was confirmed by both markers, but disagreed over the affinity of populations from west New Guinea. Mitochondrial data suggest higher connectivity among populations with fewer signals of regional substructure than microsatellite data. Considering the homogenizing effect of only a few migrants per generation on genetic differentiation between populations, marker-specific results have important implications for conservation efforts concerning this and similar species.

## Introduction

Reproductive population connectivity in spatially separated subpopulations of sessile marine species is shaped primarily through larval dispersal and mortality (Pineda et al. 2007). Larval dispersal can achieve population replenishment for exploited or depleted populations, drive colonization of new or abandoned habitats, and diversify the gene pool of isolated populations (Levin 2006; reviewed in Cowen and Sponaugle 2009). Mounting evidence for disproportionately high degrees of restricted and directed larval dispersal in many coastal and offshore species (e.g., Barber et al. 2002; Planes and Fauvelot 2002; Swearer et al. 2002; Bernardi et al. 2003; Taylor and Hellberg 2003; Ovenden et al. 2004; Baums et al. 2006; Bowen et al. 2006; Thacker et al. 2007; Schluessel et al. 2010) has revealed the potential vulnerability of demographically interdependent populations. For sessile marine species, failure of larvae to insure homogeneous population connectivity throughout the species range produces genetic population structures, which are a valuable source of information for conservation and management efforts, identifying potentially isolated or vulnerable populations and recognizing common gene flow barriers among species. In order to manage populations of marine species for commercial use or under aspects of biodiversity conservation and ecosystem functioning, baseline knowledge of their population dynamics and connectivity needs to be established (Fogarty and Botsford 2007).

The lack of congruency found in the genetic population structure of species with very similar life histories and/or larval ecology/physiology (Barber et al. 2011; DiBattista et al. 2012) highlights the need to accommodate this variability in research and management (Severance and Karl 2006). Wide geographic (distribution range) and taxonomic coverage (from intraspecific to intergeneric) in sampling of marine fauna and flora is required to develop a clearer picture of the variability inherent in these systems. This is urgently needed to counteract the steadily increasing pressure on marine resources in degrading coastal habitats (Botsford et al. 2001; Palumbi 2003; Hughes et al. 2005).

This study employs sequences of the mitochondrial control region (CR; the hypervariable D-loop; Alvarado et al. 1995) and 10 microsatellite markers to investigate the population structure of the pink anemonefish, *Amphiprion perideraion* (Bleeker 1855), across the Indo-Malay Archipelago (IMA) and one Philippines site. Nonconcordance between nuclear and mitochondrial markers is common in fish and other animals (DiBattista et al. 2012 and therein; Toews and Brelsford 2012) supporting the inclusion of both markers types for the recovery of robust phylogenetic relationships (Edwards and Bensch 2009) and to anticipate the inability of either marker to detect genetic structure, where it is present (reviewed in Karl et al. 2012).

Populations of *A. perideraion* are commercially harvested for the global marine ornamental trade, placing additional stress on population persistence in light of frequent reef demise and coastal habitat degradation (Wabnitz et al. 2003; Shuman et al. 2006). Their obligate symbiosis with sea anemones Cnidaria, Anthozoa, Hexacorallia, Actiniaria; four potential hosts) increases the risk of localized stock depletion due to commercial harvest of the hosts and host vulnerability to high temperature-induced bleaching events, projected to increase with climate change (Shuman et al. 2006; Jones et al. 2008). Although motile by nature, *A. perideraion* is sedentary, as these fish move only within the close vicinity of the sea anemone they inhabit, excluding adult migration as a factor in genetic mixing (Fautin and Allen 1997). The results will shed light on the ability of larval dispersal to connect *A. perideraion* populations on smaller and larger spatial scales.

Studies examining the population structure of *Amphiprion ocellaris*, an *A. perideraion* congener, showed strong structure across the IMA and along a known biogeographic break, the Indo-Pacific barrier (IPB; Briggs 1974) (Nelson et al. 2000; Timm and Kochzius 2008; Timm et al. 2012). This barrier, formed by the almost complete fusion of the southern Indonesian Islands chain, most recently emerged during lowered sea levels in Pleistocene glacial cycles (Voris 2000). The genetic signature of repeated isolation of Pacific and Indian Ocean populations has been detected in quite a number of marine species (e.g., Barber et al. 2002; Lourie et al. 2005; DeBoer et al. 2008; Knittweis et al. 2008; Timm and Kochzius 2008). *A. ocellaris* and *A. perideraion* share a very similar life history, demersal egg development, and a short PLD (*A. ocellaris* 8–12 days, Fautin and Allen 1997; *A. perideraion* 18 days, Wellington and Victor 1989), suggesting that geological history and restricted larval dispersal may shape the population structure of *A. perideraion* populations in a similar fashion. In addition, studies on larval recruitment of *A. perideraion* and closely related species have shown high levels of self-recruitment (*Amphiprion chrysopterus,* Beldade et al. 2012; *Amphiprion percula,* Almany et al. 2007; Buston et al. 2012; *Amphiprion polymnus*, Jones et al. 2005; *A. perideraion*, Madduppa et al. 2014), supporting expectations of a strong genetic population structure. The impact of self-recruitment and sweepstake reproduction (Hedgecock and Pudovkin 2011) can limit migrant exchange between populations, leading to demographic isolation of populations on very small spatial scales (Buston et al. 2012), although this is not always the case (Christie et al. 2010). In the search for common barriers to dispersal for purposes of conservation planning, the intergeneric comparison is of particular value and can further increase understanding of factors affecting larval dispersal.

The coral reefs of the Indo-Malay Archipelago, which support the highest global marine biodiversity (Roberts et al. 2002; Hoeksema 2007; Veron et al. 2009), are among the most threatened reef systems worldwide (Burke et al. 2002). Coastal degradation, pollution, overexploitation, and climate change all pose serious threats that require prompt action to avert irreversible damage. This region consists primarily of island states, where 350 million people live within 50 km of the coast, relying on ocean resources for their subsistence, transport, and trade (Burke et al. 2002). Results from this study can be used to further expand the knowledge base available for marine management decisions, which are currently being installed under the auspices of the Coral Triangle Initiative (CTI), a collective effort at marine resource management by Indonesia, Papua New Guinea, the Solomon Islands, Malaysia, the Philippines, and Timor-Leste (www.coraltriangleinitiative.org).

## Materials and Methods

### Sampling, DNA extraction, and amplification

With the use of SCUBA, 305 samples of *A. perideraion* were collected from 21 locations spanning the IMA and Japan. Sampling locations were chosen to transverse the IPB, to lie along the strong current of the Indonesian through flow (ITF), and to include samples from all major central and peripheral basins of the archipelago. *A. perideraion* individuals could not be found during expeditions in west Sumatra and Singapore (Batam), although these locations lie within the suggested range of this species. Fin clip samples were stored in 96% ethanol at 4°C. Genomic DNA was extracted with a commercial kit (peqGOLD Tissue DNA Mini Kit, Peqlab, Erlangen).

#### Control region

A 420-bp fragment of the D-loop segment of the mitochondrial control region was amplified with primers CR-A (TTC CAC CTC TAA CTC CCA AAG CTA G) and CR-E (CCT GAA GTA GGA ACC AGA TG) (Lee et al. 1995) for 262 individuals (from 19 locations). PCR reactions followed a standard PCR protocol detailed in Timm et al. (2008). PCR products were purified with peqGold Cycle-Pure kits (PeqLab, Erlangen). Both strands were sequenced on an ABI PRISM 310 Genetic Analyser (Applied Biosystems, Weiterstadt, Germany) after amplification with the PCR primers and the Big Dye Terminator Cycle Sequencing Kit (ver. 3.1; Applied Biosystems). *A. perideraion* sequences were subsequently deposited in GenBank.

#### Microsatellites

Primers to amplify 10 microsatellite loci for 289 individuals from 20 locations are listed and described in Table1 along with the observed and expected heterozygosities. Primers were either HEX- or FAM-labeled and used to amplify sample DNA using the protocol by Timm et al. (2012). The amplified fragments were run on an ABI 3100, using an internal 500 Rox Size Standard (Applied Biosystems). Genemarker (ver. 1.91 Demo SoftGenetics, State College, PA, USA) was used to score fragment lengths for all samples. Scoring error between runs was controlled by always including previously analyzed samples with every new 96-sample run and checking the consistency of results for these samples.

**Table 1:** Primers for the amplification of 10 microsatellite loci in *A. perideraion* with their respective motif, PCR product size, number of alleles, PCR annealing temperature, the observed (*H*_o_) and expected (*H*_e_) heterozygosities, and their biological and literature sources

Locus	Motif	Product size (bp)	No. alleles	Primers	Ann. temp. (°C)	*H*_o_	*H*_e_	Source (*Amphiprion* sp.)
Ac1578	(AC)_9_	242–266	13	F: 5′-CAGCTCTGTGTGTGTTTAATGC-3′	55.7–57	0.395	0.408	*A. clarkii* (Liu et al. 2007)
				R: 5′-CACCCAGCCACCATATTAAC-3′				
Ac626	(TC)_6_(AC)_20_	222–270	25	F: 5′-CACACATGCACACACCTTGA-3′	60	0.856	0.933	*A. clarkii* (Liu et al. 2007)
				R: 5′-TAATTGAGGCAGGTGGCTTC-3′				
Ac137	(AC)_19_	274–334	31	F: 5′-GGTTGTTTAGGCCATGTGGT-3′	55.7	0.828	0.945	*A. clarkii* (Liu et al. 2007)
				R: 5′-TTGAGACACACTGGCTCCT-3′				
CF42	(TCTG)_18_	253–453	45	F: 5′-TGCAATTATGCACCTG-3′	58.6	0.811	0.960	*A. percula* (Buston et al. 2007)
				R: 5′-TGGCCAGATTGGTTAC-3′				
CF27	(TCTA)_16_	185–229	12	F: 5′-AAGCTCCGGTAACTCAAAACTAAT-3′	60	0.794	0.881	*A. percula* (Buston et al. 2007)
				R: 5′-GTCATCTGATCCATGTTGATGTG-3′				
55	(GT)_16_	435–465	11	F: 5′-TTAACTTCCACACCCAGTCT-3′	58.7	0.712	0.798	*A. polymnus* (Quenouille et al. 2004)
				R: 5′-ACGCTGTGAGAGTCCATTAT-3′				
44	(GT)_13_	213–265	25	F: 5′-TTGGAGCAGCGTACTTAGCT-3′	58.7	0.906	0.905	*A. polymnus* (Quenouille et al. 2004)
				R: 5′-AGATGTGTTTACGCACGCTT-3′				
61	(GT)_49_	262–372	41	F: 5′-TGAACACATAAACGCTCACTCAC-3′	58.7	0.824	0.952	*A. polymnus* (Quenouille et al. 2004)
				R: 5′-AAGACAATGCCTCCACATATCTA-3′				
120	(GT)_18_N_20_(GT)_14_	454–492	19	F: 5′-TCGATGACATAACACGACGCAGT-3′	68	0.861	0.852	*A. polymnus* (Quenouille et al. 2004)
				R: 5′-GACGGCCTCGATCTGCAAGCTGA-3′				
Ac915	(AC)_9_	208–234	12	F: 5′-TTGCTTTGGTGGAACATTTGC-3′	57	0.714	0.728	*A. clarkii* (Liu et al. 2007)
				R: 5′-TCTGCCATTTCCTTTGTTC-3′				

### Data analysis

#### Control region

Consensus sequences, produced by editing of forward and reverse sequence strands in Seqman (ver. 4.05, DNAStar, Madison, WI, USA) were aligned with Clustal W (1000 bootstraps, minimal manual adjustment of indels) (Thompson et al. 1994) as implemented in BioEdit (ver. 7.0.0.1, Hall 1999). After inclusion of a GenBank sequence from the Solomon Islands (DQ343940.1, Santini and Polacco 2006), sequences were trimmed to shortest common sequence length, creating a 382 bp alignment of 263 sequences used for all subsequent analyses.

To insure suitability for population genetic analyses, the neutrality of the marker was evaluated on the basis of Tajima's D (Tajima 1989, 1993) and Fu's F_S_ (Fu 1997), which also allows the detection of a recent population expansion or bottleneck. Chakraborty's test of amalgamation (Ewens 1972; Chakraborty 1990) was included to detect potential sample heterogeneity. All tests were carried out in DnaSP (ver.5.0, Librado and Rozas 2009). A sequence of *Amphiprion akallopisos*, a closely related species, was added to allow for rooting of the genealogy.

Unless otherwise stated, all following analyses were carried out with Arlequin (ver. 3.1, Excoffier et al. 2005). Nucleotide and haplotype diversities for all populations were calculated according to Nei (1987). Overall genetic population structure in the dataset (Φ_ST_) and pairwise population differentiation (pairwise Φ_ST_) were determined with an analysis of molecular variance (AMOVA). Corresponding *P*-values for pairwise computations were adjusted to control for the false discovery rate (FDR) according to Benjamini and Hochberg (1995) (multtest, R package 2.9.0). Groups for hierarchical AMOVA testing were chosen to represent regional assemblages and/or to reflect gene flow barriers detected in pairwise population comparisons. Interpretation of significant pairwise population differences was expected to add scale to the extent of local or regional population differentiation, otherwise masked by the rather broad groupings achieved in a hierarchical AMOVA. Population groupings that provided the highest significant between-group differences were applied to test for significant differences in nucleotide and haplotype diversities among these groups using a two-tailed *t*-test (www.graphpad.com/quickcalcs/ttest1.cfm).

**Figure 1:**
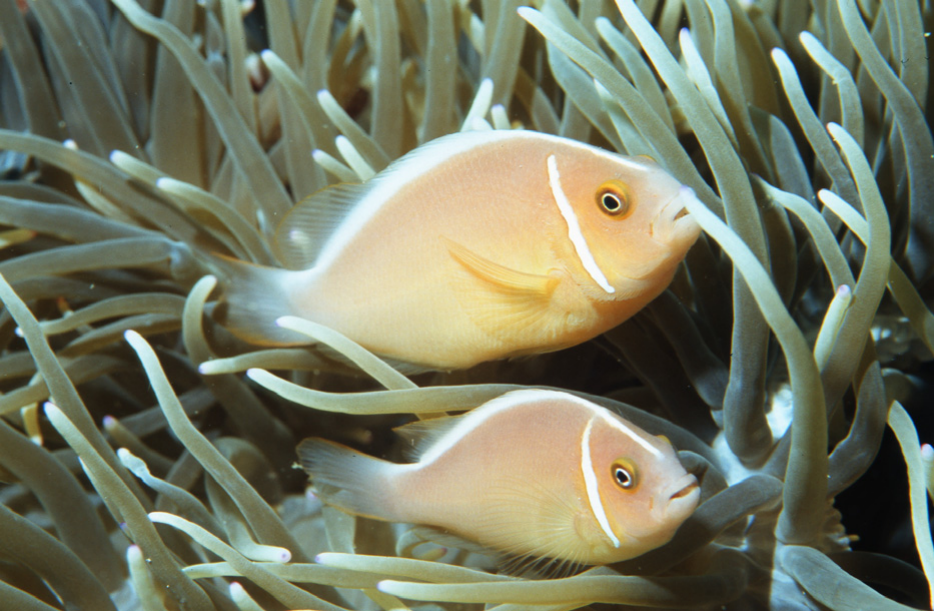
A pair of the pink anemonefish, *Amphiprion perideraion*, in *Heteractis crispa*, one of its four potential sea anemone hosts.

All haplotypes (*n* = 171) were included in the construction of a minimum spanning tree (MST) (Kruskal 1956; Prim 1957). Clades were defined as containing less mutational steps within, than between clades. Single outlier haplotypes were not defined as clades, as their position in the MST is questionable and may only be resolved with additional data. The relative frequency of clades at each location is visualized in Figure1B with pie charts imposed onto a map of the sampling area.

#### Microsatellites

The suitability of the microsatellite loci for population genetic analysis in *A. perideraion* was evaluated prior to inclusion, as none of the loci had previously been isolated and tested for this species. The expected and observed heterozygosities of loci in each population and overall were resolved in Arlequin, testing for significant deviations from Hardy–Weinberg equilibrium in the distribution of alleles. A likelihood-ratio test was used to detect linkage disequilibrium between pairs of loci (Excoffier and Slatkin 1998). *P*-values were corrected according to Benjamini and Hochberg (1995), accounting for the FDR. Loci were assessed with Microchecker (ver. 2.2.3; Van Oosterhout et al. 2004) to check for null alleles and large allele dropout.

The program FSTAT (ver. 2.9; Goudet 1995) was used to determine the mean gene diversity and allelic richness in each population. The differentiation index D (Jost 2008) was calculated with DEMEtics (ver. 0.8-5 R package; Gerlach et al. 2010) to detect average overall (mean *D*_est_) and interpopulation (pairwise mean *D*_est_) genetic differentiation in the dataset (Gerlach et al. 2010). The significance of the detected differentiation was described by *P*-values, estimated from bootstrap resampling (1000), and corrected for the FDR. The inability of *F*_ST_ to accurately reflect population differentiation when diversity within populations is high (as with polymorphic microsatellites) has been repeatedly discussed and confirmed (reviewed in Meirmans and Hedrick 2011). *F*_ST_ is expected to detect significant structure when present, but fails to rank gene flow scenarios correctly (Gerlach et al. 2010). This has led to new indices, such as the derivative of Jost's D employed here. Overall *F*_ST_ and pairwise population *F*_ST_ values have also been computed and can be found in the Supplementary Materials. A Mantel's test was conducted between the pairwise *F*_ST_ and pairwise mean *D*_est_ values to investigate the correlation computed for all population pairs. Results are in the Supplementary Material.

A hierarchical AMOVA was run for several different scenarios of population groupings. Inferences drawn from pairwise distance calculations were used for grouping decisions, although only a fraction of all tried groupings is presented here. The population structure within and among *A. perideraion* populations was further investigated with the model-based clustering method implemented in STRUCTURE (ver. 2.3.3.; Pritchard et al. 2000). The model applies a Bayesian likelihood approach to estimate the probability of correctly dividing all genotypes in the dataset among *k* number of clusters. All 289 individuals were additionally labeled according to sampling location, so that the LOCPRIOR admixture model could be applied (Hubisz et al. 2009). No information about the geographic distance between sampling locations was included. The burn-in period was set to 120,000 with 300,000 repetitions after burn-in. Each *k* (1–10 clusters) was run for 10 iterations, and probabilities were calculated on the basis of the median estimated ln probability of the data. The proportion of samples assigned to different clusters at each sampling location was visualized by means of pie chart diagrams, superimposed on the map of the sampling area. An artificial *q*-value threshold difference (≥0.25) was enforced for a clear assignment of samples to one of the proposed groups. When this value could not be reached, samples were treated as potential descendants of mixed ancestry and marked as such in the corresponding pie charts.

#### Control region and microsatellites

Geographic distances represent the shortest connection via marine pathways using a Google Earth function. The Isolation by Distance Web Service (Jensen et al. 2005) was then used to assess the correlation between geographic and genetic distance (pairwise Φ_ST_ and *D*_est_) of sampled populations by applying a Mantel's test, providing a one-tailed *P*-value for significance of the matrix correlation and the corresponding R-square. A third matrix component was added to include information on whether population pairs stemmed from the same (score = 0), adjoining (score = 1), or nonadjoining (score = 2) “discreet clusters of (genetic) exchange” according to divisions proposed by Kool et al. (2011, individual-based biophysical dispersal model). Their model simulates larval dispersal to and from recorded coral reefs in the Indo-West Pacific with the resulting patterns suggesting barriers to dispersal and identifying areas of pronounced admixture. In terrestrial ecology, the inclusion of dispersal-retarding features, such as roads or fences, in otherwise continuous landscapes, is a common addition to calculations assessing the effect of geographic isolation on genetic distances. The geographic complexity of the IMA and previous research on related species suggest that a simple isolation by distance pattern will not apply here, but may become apparent if dominant gene flow barriers are included in the calculation.

## Results

### Control region

*C*ontrol region sequences (262 individuals from 19 locations) were successfully amplified and subsequently deposited in GenBank (Table S1).

### Neutrality testing

#### Control region

**Figure 2:**
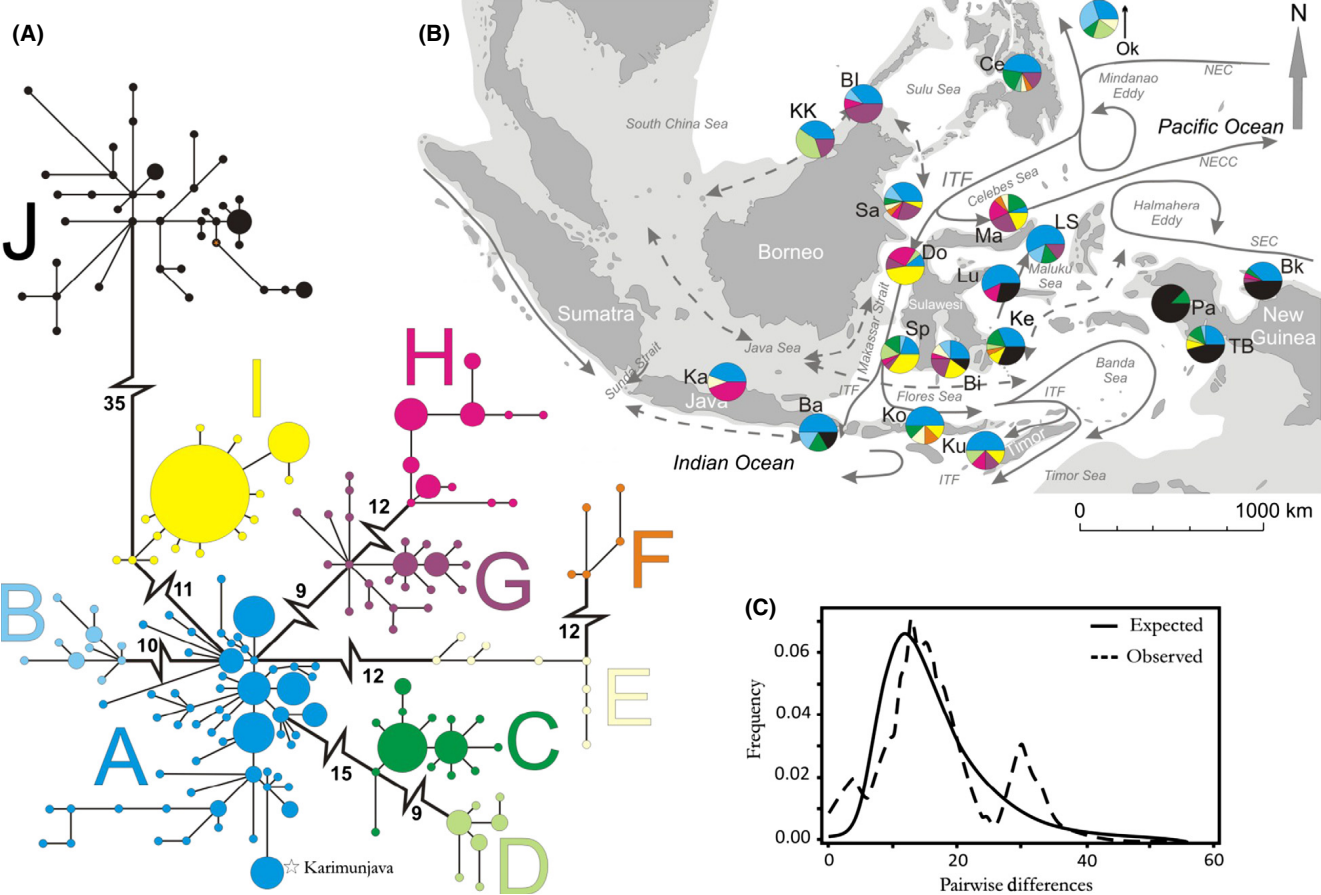
All haplotypes identified in 262 CR sequences of *A. perideraion* were used to (A) construct a minimum spanning tree (MST) divided into 10 clades (A-J), to (B) map the fractional contribution of the defined clades to populations at 19 sampling sites within the Indo-Malay Archipelago, and to (C) display the observed and expected frequencies of pairwise differences (mismatch distribution) for all haplotypes under a model of sudden population expansion. The size of circles in A is relative to the number of individuals represented by that haplotype, with the smallest circle constituting one and the largest circle 12 individuals. The length of connections between haplotypes is relative to the number of mutational steps between the two (shortest connection represents one mutation), except for connections between clades, where the number of unsampled mutational steps is given. For the map shown in B, major surface currents are indicated with arrows (dashed arrows depict seasonally reversing currents). Dark gray areas are present-day land formations, and light gray shading indicates marine habitat exposed during the Pleistocene glacial maxima, which led to a 120 m drop in sea level (Voris 2000).

A nonsignificant test outcome for Tajima's D failed to reject the neutrality of the marker and confirmed its suitability for further analysis (Table2). Fu's F produced a large negative and significant test statistic (considered significant at the 2% level), implicating departures from population equilibrium (e.g., population expansion). The mismatch distribution (Fig.2C) supported this result by describing a predominantly unimodal curve of pairwise haplotype differences, expected under a model of sudden population expansion (Rogers and Harpending 1992). Both the sum of squared deviations and Harpending's raggedness index showed no significant deviation from a model of sudden demographic expansion (Table2). The presence of two additional small peaks may underline substructures in the dataset (Ray et al. 2003), but they persisted when regional assemblages were analyzed. Chakraborty's test of amalgamation was significant, supporting a scenario of amalgamation of previously separated populations, as the neutrality of the marker was established with other tests (Table2).

**Table 2:** Results for several statistical tests to evaluate the neutrality of the marker (mitochondrial control region). Values in bold are considered significant

Neutrality tests		
Tajima's D	−0.979	*P* > 0.1
Fus FS	−**23.64**	*P* < 0.05
Chakraborty's test	**0.007**	*P* < 0.05
Mismatch distribution		
SSD	0.0031	*P* > 0.1
Raggedness index	0.0008	*P* ≫ 0.1

### Microsatellite characterization and testing

#### Microsatellites

Heterozygosities were high (0.714–0.906) in all loci except AC1578 (0.395). The values for observed and expected heterozygosities were close, but observed heterozygosities tended to be lower in most cases (Table1). The highest numbers of alleles were found in loci CF42 and 61, with 45 and 41 alleles, respectively. These two loci also had the most frequent suspected occurrence of null alleles among all populations. Evidence of null alleles was found in 42% of tested populations for locus CF42 and in 32% for locus 61. Both loci displayed a larger number of population-specific deviations from HWE than other loci, which further supports suggestions of null allele presence (data not shown). Null alleles are expected to falsely inflate genetic differentiation of populations. However, the overall Φ_st_ remained unchanged (increased by 0.003) when both loci were excluded. Therefore, these loci where included in the further analysis.

None of the tested populations showed a consistent deviation from HWE across loci. As a consequence, a gross violation of model assumptions for ideal populations (random mating, no mutation, no drift, no migration) is unlikely, and the scale of sampling appears to capture discrete populations (Johnson and Black 1984).

Three different loci combinations (44-CF27, Ac626-CF27, and Ac137-CF42) indicate linkage disequilibrium in three different populations (Do, Bk, and TB). If markers are truly linked, this linkage is expected to carry across populations, which was not the case here. Therefore, all loci were expected to assort independently.

### Genetic diversity

#### Control region and microsatellites

Haplotype (*h *=* *0.81–1.00) and nucleotide diversities (*π *= 0.037–0.078) were consistently high among populations, being lowest in Karimunjava (Table3). The population in Karimunjava also had the lowest allelic richness and second to lowest gene diversity. Nucleotide and gene diversities were highest in New Guinea and in populations lining the eastern Banda Sea. The nucleotide diversity of the New Guinea group (hierarchical AMOVA [Bk, TB, Pa], Table4) in CR data was significantly higher (unpaired *t*-test: *t* = 2.121, df = 14, *P *=* *0.0281) than in the rest of the archipelago. Most populations located at the northern (Ce, Ok, BI), western (Ka), and southern peripheries (Ku) of the sampling area had nucleotide diversities at the lower end of the spectrum, potentially suggesting that founder events with subsequent expansion may have shaped the diversity of these populations. Allelic richness reflects the same pattern found in gene diversity (Table3).

**Table 3:** Sample sites for *A. perideraion* samples collected from across the IMA with the respective abbreviations (Abbr.) and regional placement. The number of individuals (*N*_ind_) analyzed per location for each dataset (CR and Msat) is indicated. Both datasets are composed of the same individuals, with differences in the number of individuals indicating that samples in addition to those constituting the other dataset were incorporated. For the CR dataset, the number of haplotypes (*N*_haplo_), the ratio of haplotype number to total individuals sampled (*N*_haplo_/*N*_ind_), the haplotype (*h*) and nucleotide (*π*) diversities are given per site. Msat data are described with gene diversity and allelic richness, including their respective standard deviations (SD)

Sample sites	Region	Abbr.	Control region-D-loop (CR)					Microsatellites-10 loci (Msat)		
			*N*_ind_	*N*_haplo_	*N*_haplo_/*N*_ind_	*h *+ SD	*π *+ SD	*N*_ind_	Gene diversity ± SD	Allelic richness ± SD
Spermonde	SW Sulawesi	Sp	20	13	0.65	0.95 ± 0.028	0.054 ± 0.028	29	0.798 ± 0.417	3.19 ± 0.55
Donggala	NW Sulawesi	Do	19	14	0.74	0.96 ± 0.031	0.043 ± 0.023	25	0.79 ± 0.414	3.15 ± 0.58
Manado	NE Sulawesi	Ma	16	14	0.88	0.98 ± 0.028	0.053 ± 0.028	24	0.826 ± 0.432	3.21 ± 0.53
Lembeh Strait	NE Sulawesi	LS	7	7	1.00	1.00 ± 0.076	0.046 ± 0.027	8	0.772 ± 0.427	3.09 ± 0.8
Luwuk	E Sulawesi	Lu	14	11	0.79	0.97 ± 0.037	0.066 ± 0.034	14	0.837 ± 0.444	3.24 ± 0.6
Bira	S Sulawesi	Bi	20	19	0.95	0.995 ± 0.018	0.062 ± 0.032	21	0.801 ± 0.421	3.19 ± 0.66
Kendari	E Sulawesi	Ke	19	18	0.95	0.99 ± 0.019	0.078 ± 0.04	18	0.839 ± 0.441	3.24 ± 0.57
Sangalaki	E Borneo	Sa	17	16	0.94	0.99 ± 0.023	0.047 ± 0.025	19	0.826 ± 0.434	3.21 ± 0.65
Karimunjava	off N Java Coast	Ka	9	5	0.56	0.81 ± 0.12	0.037 ± 0.021	8	0.727 ± 0.400	2.8 ± 0.66
Bali	S Bali	Ba	6	6	1.00	1.00 ± 0.096	0.066 ± 0.039	7	0.815 ± 0.455	3.16 ± 0.66
Komodo	Komodo/Flores	Ko	8	8	1.00	1.00 ± 0.063	0.049 ± 0.028	10	0.721 ± 0.392	3.05 ± 0.66
Kupang	Timor	Ku	8	8	1.00	1.00 ± 0.063	0.044 ± 0.025	10	0.777 ± 0.420	3.11 ± 0.75
Banggi Islands	N Borneo	BI	11	11	1.00	1.00 ± 0.039	0.042 ± 0.023	11	0.824 ± 0.441	3.23 ± 0.68
Kota Kinabalu	N Borneo	KK	5	5	1.00	1.00 ± 0.127	0.057 ± 0.035	5	0.849 ± 0.481	3.27 ± 0.53
Biak	E New Guinea	Bk	22	19	0.86	0.97 ± 0.028	0.072 ± 0.037	23	0.83 ± 0.434	3.19 ± 0.52
Cebu	Philippines	Ce	19	16	0.84	0.98 ± 0.027	0.045 ± 0.023	17	0.806 ± 0.426	3.17 ± 0.574
Okinawa	Japan	Ok	10	10	1.00	1.00 ± 0.045	0.044 ± 0.024	0	na	na
Misool	Maluccas	Mi	0	na	na	na	na	2	na	na
Pisang	W New Guinea	Pi	0	na	na	na	na	3	na	na
Papisol	W New Guinea	Pa	0	na	na	na	na	13	0.859 ± 0.455	3.29 ± 0.46
Triton Bay	W New Guinea	Tr	0	na	na	na	na	22	0.825 ± 0.436	3.26 ± 0.53

**Table 4:** Hierarchical AMOVA groupings of *A. perideraion* populations in the Indo-Malay Archipelago based on pair-wise distances of mitochondrial control region sequences (Φ values) and 10 microsatellite loci (*F* values). Bold values describe the highest index support for the tested combinations

Groupings	Control region-D-loop		Microsatelites-10 loci	
	Φ_CT_	*P* ± SD	*F*_CT_	*P* ± SD
No groups	0.138	<0.000 ± 0.000	0.015	<0.000 ± 0.000
2 Groups				
[Ka][all others]	0.0356	0.221 ± 0.015	0.0417	0.056 ± 0.007
[Bk][all others]	0.0789	0.055 ± 0.007	0.0068	0.094 ± 0.009
3 Groups				
[Bk,Mi][Ka][all others]			**0.0181**	**0.003 ± 0.002**
[Bk,Pa,TB][Ka][all others]	**0.1985**	**<0.000 ± 0.000**	0.0134	0.002 ± 0.001
[Bk,Pa,TB,Ke][Ka][all others]	0.1857	<0.000 ± 0.000		
[Bk,Pa,TB][Ka,Ba][all others]	0.1768	<0.000 ± 0.000		
4 Groups				
[Bk][Pa,TB][Ka][all others]	0.1831	<0.000 ± 0.000	0.0099	0.018 ± 0.004
[Bk,Pa,TB][Lu;Ke][Ka][all others]	0.1764	<0.000 ± 0.000		
[Bk,Mi][Pi][Ka][all others]			0.0180	0.002 ± 0.001
[Bk,Mi][Ce][Ka][all others]			0.0147	0.005 ± 0.002
[Bk,Pa,TB][Lu;Ke,LS][Ka][all others]	0.1616	<0.000 ± 0.000		

The ratio of the number of haplotypes found to the number of sampled individuals from populations was high overall, with the lowest ratio seen in Karimunjava (0.56) (Table3). Typical for a control region dataset, the fraction of singleton haplotypes found in populations was high, accounting for 100% of private haplotypes in 13 of the 19 populations analyzed (data not shown). Only populations in Manado, Donggala, and Spermonde, situated prominently along the ITF, contained more shared than private haplotypes.

### Overall genetic structure

#### Control region and microsatellites

AMOVAs with the CR and Msat datasets showed highly significant deviations from panmictic population conditions (Φ_ST_ = 0.096, *P* < 0.0001; mean *D*_est_ = 0.17; *F*_ST_ = 0.015, *P* < 0.0001). Despite the low sample size from Karimunjava, the hierarchical AMOVAs for both datasets best supported a differentiation of Karimunjava from the central archipelago and eastern populations, as well as an isolation of eastern populations from the center. However, the CR dataset finds the highest between-group variation when the eastern group includes east and west New Guinea populations (Bk, Pa, TB) (Φ_CT_ = 0.199, *P* < 0.000), while the Msat dataset best supports a smaller eastern group with only Biak (and Misool), grouping Papisol and Triton Bay with the central populations (*F*_CT_ = 0.018, *P* = 0.003). A summary of the examined group configurations is provided in Table4. Both datasets support a scenario of central mixing, with pronounced western and eastern population differentiation.

Testing for the effect of isolation by distance produced a very weak correlation (*r* = 0.199, *P* = 0.05) between geographic and genetic distance with Msat data and no significant correlation with the CR dataset. *R*-squared values were very low (explaining <10%), and the regression lines did not describe the spread of the data. Controlling for the effects of potential dispersal-retarding current features, as modeled by Kool et al. (2011), with a third matrix component, did not reveal masked IBD in either dataset.

### Regional structures

#### Control region and microsatellites

**Figure 3:**
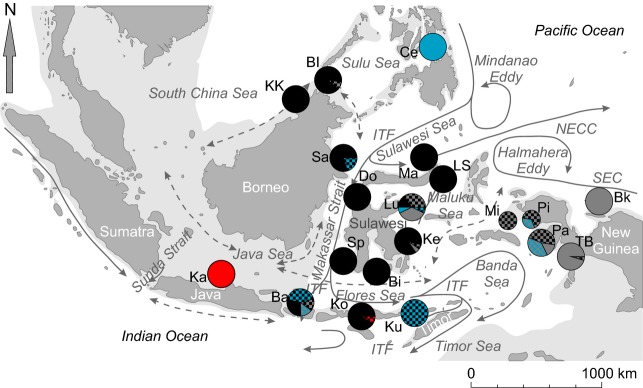
Map of the study area with pie charts depicting the fractional assignment of *A. perideraion* individuals from each sampling location to one or more of the four (k = 4) genotype clusters defined by STRUCTURE (ver. 2.2., Pritchard et al. 2000), based on 10 microsatellite loci. Red, blue, black, or gray pie slice colorations represent one of the four clusters each. Checkered pie slices depict potential scenarios of mixed ancestry of the two colors used for the pattern. This was applied when a threshold value difference (≥0.25) between two alternative probabilities of group assignments could not be reached.

Both marker systems revealed extensive regional population substructure in the IMA with patterns in opposition to a simple IBD model and not adhering to dynamics expected from the impact of dominant ocean currents (e.g., ITF) on larval transport. Pairwise population differentiation measures (pairwise Φ_ST_ and mean *D*_est_) are listed in Table5 for all populations, with significance adjusted to control for the false discovery rate. Considering only locations with data available from both datasets, the Msat dataset revealed 72 instances of significant population differentiation, as opposed to 29 in the CR dataset. Both datasets agreed on the pronounced differentiations of Biak (range *D*_est_ = 0.111 [Sp] – 0.373 [Ka], Φ_ST_ = 0.169 [Pa] – 0.267 [Do]) and Karimunjava (range *D*_est_ = 0.17 [Mi] – 0.401 [Sa], Φ_ST_ = 0.109 [Bi] – 0.589 [Pa]) to the rest of the populations, while only the Msat dataset indicated differentiation of the Philippine population (range *D*_est_ = 0.106 [Sp] – 0.373 [Ka]) (Table5, Fig.3).

**Table 5:** Population pairwise differences in control region sequences (Φ_ST_ index, above diagonal) and microsatellite data (*D*_est_ index, below diagonal) for *A. perideraion* for all sampling sites are shown (1000 permutations). Bold values denote significance at *P* ≤ 0.05 (1000 bootstraps) after correction for multiple testing (Benjamini and Hochberg 1995, False Discovery Rate procedure). Corresponding *F*_ST_ index values for the Msat dataset is available in Supplementary Material, Table S2

	Sp	Do	Ma	LS	Lu	Bi	Ke	Sa	Ka	Ba	Ko	Ku	BI	KK	Bk	Ce	Pa	TB	Ok
Sp		0.022	0.035	0.047	**0.113**	0.035	**0.093**	**0.07**	**0.16**	0.051	0.025	0.028	**0.135**	0.015	**0.217**	0.049	**0.538**	**0.171**	0.049
Do	**0.071**		0.044	0.155	**0.161**	0.034	**0.152**	**0.101**	**0.192**	**0.174**	0.085	0.117	**0.155**	**0.167**	**0.267**	**0.139**	**0.594**	**0.222**	**0.182**
Ma	0.062	0.032		0.042	**0.106**	0.016	**0.104**	0.012	**0.126**	0.075	0.013	0.051	0.038	0.048	**0.217**	0.029	**0.541**	**0.180**	0.087
LS	**0.116**	**0.146**	0.101		0.043	0.005	0.067	−0.029	**0.154**	−0.065	−0.029	−0.057	0.01	−0.016	0.165	−0.031	**0.554**	0.147	−0.025
Lu	**0.096**	**0.115**	**0.123**	**0.139**		0.022	−0.018	0.074	0.138	−0.051	0.051	0.045	0.099	0.095	0.001	**0.095**	**0.325**	0.020	**0.093**
Bi	0.044	**0.068**	0.030	0.06	**0.14**		0.044	0.000	**0.109**	−0.003	−0.011	0.009	0.023	0.067	**0.127**	**0.037**	**0.453**	**0.116**	0.053
Ke	**0.073**	0.062	0.056	0.023	**0.109**	0.002		**0.103**	**0.155**	−0.035	0.058	0.079	**0.14**	0.082	0.000	**0.098**	**0.236**	−0.016	0.083
Sa	0.036	**0.073**	0.070	**0.141**	**0.14**	0.037	0.051		**0.132**	0.025	−0.033	0.001	−0.01	0.058	**0.206**	−0.011	**0.558**	**0.185**	0.035
Ka	**0.339**	**0.299**	**0.258**	**0.316**	**0.296**	**0.299**	**0.328**	**0.401**		**0.143**	**0.115**	**0.148**	**0.176**	0.182	**0.242**	**0.163**	**0.589**	**0.203**	**0.165**
Ba	**0.161**	**0.205**	**0.151**	0.081	0.109	0.109	0.105	0.093	**0.359**		−0.01	−0.016	0.078	0.024	0.027	0.012	0.395	0.022	−0.01
Ko	0.088	**0.145**	**0.111**	0.03	**0.118**	**0.122**	**0.133**	**0.147**	**0.276**	**0.154**		−0.035	0.053	0.044	0.182	−0.026	**0.548**	0.152	0.004
Ku	**0.109**	0.122	**0.197**	0.094	0.038	**0.162**	**0.128**	**0.11**	**0.305**	**0.266**	**0.163**		0.049	0.014	**0.179**	0.007	**0.578**	**0.163**	0.005
BI	0.031	**0.146**	0.041	0.085	**0.113**	0.027	−0.000	0.055	**0.367**	0.056	**0.137**	**0.113**		0.098	**0.219**	0.046	**0.586**	**0.209**	**0.114**
KK	0.001	0.099	0.067	0.029	0.062	0.086	−0.038	0.005	**0.385**	0.114	0.027	0.032	−0.100		0.2	0.006	**0.545**	0.149	−0.013
Bk	**0.111**	**0.204**	**0.164**	**0.131**	**0.12**	**0.166**	**0.135**	**0.139**	**0.373**	**0.188**	**0.203**	**0.161**	0.102	0.103		0.197	**0.169**	−0.124	**0.203**
Ce	**0.106**	**0.115**	**0.184**	**0.223**	**0.109**	**0.162**	**0.145**	0.048	**0.37**	0.128	**0.214**	0.067	**0.117**	**0.177**	**0.165**		**0.566**	−0.01	0.002
Pa	0.081	**0.132**	**0.119**	0.086	0.102	0.082	0.077	0.077	**0.362**	0.070	**0.136**	0.043	0.075	0.046	0.080	**0.127**		0.133	**0.555**
TB	**0.073**	**0.124**	0.078	**0.128**	0.057	0.078	0.042	0.079	**0.312**	**0.213**	**0.136**	**0.119**	0.088	−0.077	0.023	**0.178**	0.090		**0.163**

Further evidence for the absence of a simple IBD dispersal mechanism can be seen in the many significant pairwise differences between proximate locations. Donggala, exposed to the strong currents of the ITF in the Makassar Strait, shows a surprisingly high number of significant differentiations to other populations (CR – 11 populations, Msat – 14 populations). Among them are the two most proximate upstream and downstream locations in the datasets, Sangalaki and Spermonde. A similar situation is seen in Luwuk, which is significantly different from its closest northern (LS) and southern (Ke) neighbors in the dataset (Msat data only), and in Kupang and Komodo, which are significantly different from one another, as well as to samples from Bali and populations just to the north (Bi, Ke).

### Minimum spanning tree and clade distribution

#### Control region

The minimum spanning tree (MST) shown in Figure1A divides the dataset into ten clades, which are separated by 9–35 nucleotide substitutions (ns). Connections between clades are not drawn to scale, but instead ns separating clades are shown. Connections within clades are drawn to scale.

Clade A holds the central position in the star-like topography of the tree, with all other clades directly or indirectly diverging from it. Due to its central position and its presence in all sampled populations (except Papisol) (Fig.2), this clade is most likely to contain ancestral haplotypes. The assumed most ancestral (most central and shared) haplotypes of clade A stem from eastern (Ma, Bk, Lu) and southern populations (Bi, Sp) (Fig.2). The population from Okinawa forms a northern exception here, as it too contains haplotypes of the central clade A. All four individuals from Karimunjava that were placed within clade A carry the same haplotype, which lies on a peripheral, terminal branch, ten ns from the next-closest clade A haplotype.

Most of the sampled populations were found to contain clade C and/or clade D haplotypes, the exceptions being Karimunjava, Luwuk, Bira, and the Banggi Islands. Clade E and clade F haplotypes were found in northeastern populations (Ce, Ok), along the Celebes Sea (Ma, Sa) and in some of the populations lining the Java and Flores Seas (Ka, Bi, Ko, Ke), but were absent from New Guinea, the Banda and Maluku Seas, the Makassar Strait, and the South China Sea. Both clades contain only singleton haplotypes with internal divergences of up to nine ns.

Clade G haplotypes are found throughout most of the IMA although the haplotype from its most southern expansion in Kupang is 11 ns removed from the next-closest clade member. A similar situation is found in clade H, where the haplotype from Kupang diverges by ten mutational steps. The high number of ns for these and other outliers makes their direct clade association questionable. However, within the given dataset, no alternative connections were suggested by the analysis. Clade H is dominated by haplotypes from Karimunjava, Donggala, and Manado, with Karimunjava at the clades' most basal position.

The smallest divergence between haplotypes (mostly 2–3 ns) is found in clade I, which is predominantly found along the northern, western, and southern coastline of Sulawesi and in other populations situated along the ITF. Moving into and across the Banda Sea, the presence of clade I haplotypes decreases, while that of clade J haplotypes increases (Fig.2B). The latter is confined to Bali, east Sulawesi, and New Guinea and is by far the most divergent clade, removed by 35 ns. Nevertheless, in a phylogeny with its sibling species, *A. sandaracinos* and *A. akallopisos*, these haplotypes clearly group with *A. perideraion* (data not shown). Clade J also includes the haplotype from the Solomon Islands, which was included from GenBank. Haplotypes from Biak and the western New Guinea populations are distributed throughout this clade, occupying both central and very divergent peripheral positions.

### STRUCTURE analysis

#### Microsatellites

Bayesian likelihood analysis implemented in STRUCTURE suggests that a division of the dataset into four clusters (*k *=* *4) is most probable (Fig. S1, Supplementary Materials). Karimunjava consists exclusively of red cluster genotypes, which are only again detected as a small fraction in Komodo with genotypes of potential mixed ancestry (red/black checkered fraction of pie chart, Fig.3). The Philippine samples are similarly characterized by consisting only of pure blue cluster genotypes, while here the connectivity to the rest of the archipelago is still visible with primarily mixed blue genotypes (exception in Luwuk) detected in central, eastern, and southern populations, although completely absent from the Makassar Strait (Sp, Do) and northern Sulawesi populations (Ma, LS). The black cluster is confined to the central IMA (Sulawesi, Bali, Komodo) and to sampling locations on the north and east coast of Borneo (KK, BI, Sa). Moving up the east coast of Sulawesi, black cluster members are displaced by gray cluster members and samples, suggesting a combined black, gray and blue (Philippine cluster) ancestry. Crossing the Banda Sea, only samples of potential gray/black mixed ancestry remain and no pure black genotypes can be found. Samples collected at the most eastern locations, Bk (northeast New Guinea) and TB (central west New Guinea), are all members of the gray cluster, with only one sample of mixed or unclear ancestry in TB (blue/black).

The overall pattern suggests (1) a genetic break between the Java Sea population (Karimunjava) and all other locations, (2) a unidirectional (north to south) connectivity of the Philippines to the rest of the archipelago, (3) mixing of central populations along the ITF and within and across the Sulu Sea, and (4) a gradual eastern displacement of the black cluster genotypes by gray genotypes.

## Discussion

### Restricted gene flow across the IMA

The present study used 10 microsatellite loci and sequence data of the mitochondrial CR to investigate the population structure of *A*. *perideraion* in the IMA. Potential barriers to gene flow acting on the sampled populations were identified, and the found structure was placed in its historic and phylogenetic context. The study found substantial population structure overall for both marker types (Φ_ST_ = 0.096, *P* < 0.0001; mean *D*_est_ = 0.17; *F*_ST_ = 0.015, *P* < 0.0001), confirming expectations derived from genetic population structuring seen in other anemonefish (*A. ocellaris*, Nelson et al. 2000; Timm et al. 2012) and other reef-dwelling species with a pelagic larval phase (e.g., Bay et al. 2004; DeBoer et al. 2008; Leray et al. 2010; reviewed in Carpenter et al. 2011). Demersal egg development (Riginos et al. 2011), a relatively short PLD (18 days) (Wellington and Victor 1989), site attachment of adult fishes (Fautin and Allen 1997), and high rates of self-recruitment (Madduppa et al. 2014) are all expected to contribute to the observed structure and highlight the vulnerability of this and similar species from a conservation standpoint.

### Population structure and genetic diversity within the IMA

Population breaks between eastern, central, and western IMA populations detected in this study mirror similar breaks in a congener of *A. perideraion* (*A. ocellaris*, Timm and Kochzius 2008; Timm et al. 2012). Hierarchal AMOVA found that the highest significant genetic differentiation between regional groups is achieved when Karimunjava and Biak (with Misool) form western and eastern groups, respectively, although disagreement among markers exists in assigning west New Guinea populations to the central or the eastern group. In addition, both marker types showed a large number of significant pairwise differences between populations not adherent to a simple isolation by distance model or following prominent oceanographic features (e.g., ITF). Significant population differentiation between geographically proximate locations, for example, along and across the Makassar Strait (*D*_est_ = 0.071–0.073, Table5), across and along the Flores Sea (*D*_est_ = 0.154–0.266), and along other coastlines could indicate that barriers to gene flow are acting on these populations. The analysis revealed significant substructures within the IMA and barriers to gene flow that may need to be considered for conservation purposes.

Amalgamation of previously isolated and secondarily admixed divergent gene pools, which is characteristic for populations in highly fragmented and repeatedly fused habitats produced during Pleistocene glaciations, is here supported by a significant Chakraborty's test of amalgamation and a large negative Fu's F (Table2). Excluding the population from Karimunjava, high nucleotide diversities found in all locations support the proposed mechanism of amalgamation of populations previously isolated in individual basins of the IMA (McManus 1985).

Both the sum of squared deviations and Harpending's raggedness index indicate nonsignificant deviation from expectations under a simulated sudden demographic expansion model, although the additional small peaks in the observed distribution may indicate a gradual move towards demographic equilibrium (Table2, Fig.2C). Mismatch distributions for mitochondrial CR data in *A. ocellaris* at three different spatial scales (southeast Sulawesi/Sulawesi/entire IMA) also produced “trimodal” mismatch distributions at all scales (Timm and Kochzius 2008). Colonization of newly forming habitats with the gradual rise of seawater levels would explain a pattern of sudden population expansion as indicated here and as also found in other species populating the IMA.

### The eastern IMA

The hierarchical AMOVA grouped all west and east New Guinea locations (Bk, TB, Pa) with CR data, but isolates east New Guinea (Bk) (and Misool) from all other populations using Msat data. The low sample number from Misool does not allow conclusions to be drawn with any reliability about this population, but its association with east New Guinea, instead of more proximate west New Guinea locations, may give some indication that this population could be subject to other dynamics (Barber et al. 2011). Population genetic analysis with a hierarchical AMOVA of *A. clarkii* Msat data (sibling species, unpublished data) marked Misool as a divergent population, forming its own group. In *A. ocellaris*, the population from Misool grouped with other west New Guinea populations and did not appear as distinct (Timm et al. 2012). Misool's association with Pacific populations in *A. ocellaris* could not be ascertained, as the distribution of this species does not extend that far. A study by Timm et al. (2008) sets the speciation process of *A*. *perideraion* from an ancestral type well within the Pleistocene glacial oscillations, approx 1.6 mya, starting at the Pacific fringes of the IMA and within some of its basins (South China Sea, Sulu Sea, and Celebes Sea). Considering that the central position of the haplotype network is dominated by east New Guinea and Banda Sea haplotypes (Fig.2), one could speculate that CR data are showing signals of an invasion pathway of *A. perideraion* from Pacific populations into the central IMA.

The STRUCTURE analysis revealed a clear association of west New Guinea with the east of the Island, although inspection under the mixed ancestry model diffused the clear delineation, indicating increased connectivity between the southern (TB) population and Biak, and increasing connectivity across the Banda Sea moving up the west coast. Pairwise differences in the CR data identified the eastern IMA group (Bk, TB, Pa) as the most divergent population (Φ_ST_ = 0.116–0.594, Table5), also indicated by the large distance (35 ns) of black clade haplotypes (Fig.2B) dominating eastern locations. Papisol (west New Guinea) stands out as the most divergent population in this group, a trend not reflected in the Msat data where Biak (east New Guinea) produces the highest pairwise differences. Overall, Pacific populations of New Guinea should be considered separate from those lining the Banda Sea. Further sampling in Misool and the surrounding islands could clarify which mechanisms are shaping populations there.

### The western IMA

Measures of pairwise population divergence and hierarchical AMOVA also highlight the strong differentiation of the Java Sea (Ka) samples from the central and eastern IMA populations. Despite the seasonally oscillating currents (Fig.2B) that appear to be connecting the Java Sea to the central IMA, a genetic break has been detected here for quite a number of species, including several fish (Bay et al. 2004; Winters et al. 2010; Gaither et al. 2011). As present-day current patterns often fail to explain the population structure found in the IMA for species with a pelagic larval phase, common genetic signals for barriers to connectivity are often attributed to population fragmentation caused by eustatic sea level fluctuations. Extreme sea-level low stands (up to −130 m) during glacial maxima of the Pleistocene (most recently approx 20,000 ya) led to the formation of an almost uninterrupted land barrier known as the Indo-Pacific barrier (IPB) (Fleminger 1986) along the southern chain of islands that now form part of Indonesia (Fig.2B, light shading around land structures). This led to a massive reduction of the ITF and Indian and Pacific Ocean basin connectivity (Voris 2000). Vicariance, driven by repeated marine habitat reduction and fragmentation, is believed to have pushed allopatric speciation within and along the IMA, as well as enabling genetic drift to manifest itself within separated populations (McMillan and Palumbi 1995; Williams 2000; Kochzius et al. 2003). The genetic structure detected in *A. perideraion* populations may also show remnant signs of the Indo-Pacific barrier as both marker types confirm a significantly reduced gene flow between the Java Sea population and all other locations sampled in the IMA, with the exception of Luwuk (CR) and the Kota Kinabalu (Msat) site (Table5).

From a conservation standpoint, it is also important to know whether phylogeographic barriers still persist today in order to adjusted ecosystem management strategies accordingly. Model simulations by Kool et al. (2011) of larval dispersal (15–30 day PLD) in the IMA under contemporary oceanographic conditions demonstrated that larvae released in the Makassar Strait and western Flores Sea would not enter the Java Sea, thereby failing to reach Karimunjava. The predicted PLD of *A. perideraion* is only 18 days, much less than the max PLD (30 days) used for virtual larvae in the model, so that the trajectory of *A. perideraion* larvae can be expected to be even more restricted. This strengthens the case for a divergence of the *A. perideraion* population in Karimunjava even under contemporary oceanic conditions, suggesting that a continued isolation of the Java Sea population from the rest of the IMA should be considered and accounted for in management plans.

Two studies investigating the population structure of *A. ocellaris* (control region sequences Timm et al. 2008; six microsatellite loci, Timm et al. 2012) found that samples from Karimunjava were more strongly associated with more western (including Indian Ocean) locations than with the proximate Islands of Bali, Komodo, and Sulawesi, which also agrees with model predictions for this area. The Karimunjava sampling site describes the most western location where *A. perideraion* could be found so that the affinity of Karimunjava to Indian Ocean haplo- and genotypes could not be ascertained. Efforts to sample *A. perideraion* populations in the 1000 Islands Marine National Park northeast of Jakarta, in Padang on the west coast of Sumatra, and in Batam (Malakka Strait) were unsuccessful, although all three locations lie within the suggested distribution of *A. perideraion* (Fautin and Allen 1997). In comparison with other sampling locations, reduced genetic diversity for both markers, in addition to its absence at more western sites, may suggest that *A. perideraion* populations in the western expansion of the species range may be especially vulnerable to disturbance and/or exploitation. More extensive sampling in this area is needed to strengthen these conclusions.

### The northern IMA

The effect of a massive reduction in gene flow through repeated and nearly complete closure of seaways connecting the South China and Sulu Seas during the Pleistocene eustatic oscillations could not be detected in either dataset, as haplo- and genotypes in the two populations from northern Borneo did not indicate consorted divergence from the central IMA. This may indicate that the recolonization of the northern coastline of Borneo radiated from the Celebes and Sulu Seas, once the coastline became again submerged and connecting seaways reopened (at approx. 10,000 ya; Crandall et al. 2012). Analysis of the false clown anemonefish, *A. ocellaris*, population structure (CR, Timm and Kochzius 2008) produced a similar result, with no apparent emergence of a distinct South China Sea clade present in Kota Kinabalu or the Banggi Islands. Several population genetic studies dealing with invertebrates also suggest a similar invasion succession: giant clams, *Tridacna crocea* and *T. maxima* (Kochzius and Nuryanto 2008; Nuryanto and Kochzius 2009), and the blue starfish *Linckia laevigata* with its ectoparasite *Thyca crystalline* (Kochzius et al. 2009). Predictions of connectivity under contemporary oceanic conditions according to Kool et al. (2011) classify the South China Sea, Sulu Sea, and Celebes Sea as belonging to the same “discreet cluster of exchange among populations.”

According to model predictions of simulated larval dispersal, the Philippines are expected to show considerable retention of larvae, with very limited larval exchange in and out of the Sulu Sea (Kool et al. 2011). The population groupings best supported by either marker system do not, however, predict the isolation of the Cebu population, although pairwise comparisons do show a considerable amount of differentiation, including a barrier across the Sulu Sea towards the north coast of Borneo (Banggi Islands, Kota Kinabalu). STRUCTURE analysis very clearly defines the isolated status of the Philippines, corroborating model predictions and indicating the need to place special attention on the management of the coral reef fauna resident there. The special status of Philippine populations was also specified for other fish species, such as two species of seahorses (Lourie et al. 2005) and the three-spot damsel *Dascyllus trimaculatus* (Ablan et al. 2002), but was not detectable in the analysis of *A. ocellaris*, independent of the markers applied (CR or Msat) (Timm et al. 2012).

## Conclusions and Implications for Management

The population structure found in *A. perideraion* in the IMA is marked by Pleistocene isolation and persisting barriers to gene flow. Considering that even very small numbers of migrants every few generations can lead to population homogeneity and produce misleading signs of demographically relevant population connectivity, the detection of significant differentiation between populations should be taken quite seriously from a conservation standpoint. Conservation efforts for the protection of marine resources are often concerned with understanding the scale at which efforts are most effective to insure population persistence through demographic connectivity in networks of marine reserves.

The many instances of detected significant differentiation and the strong overall population structure detected with both marker systems suggest that local populations may need to be managed at a local scale, as successful intermediate or long distance dispersal could be rare. Despite the obvious need for the installation of marine reserve networks in the IMA, which are being developed in part under the management of the Coral Reef Initiative, other factors such as coastal development, climate change-induced reef demise (bleaching), and the continued degradation of coastal habitats through pollution and unchecked exploitation may override any conservation efforts. The lack of suitable settlement habitats outside of reserves can prevent an overspill effect from reserve areas and negate efforts to increase marine resources for local populations. In the case of *A. perideraion* and other anemonefish, harvesting of sea anemones removes suitable settlement habitat, preventing any larvae from settling. Without management strategies targeting vital components of the life history of these and other fishes, reserves may solely act as “islands of bounty” in an otherwise desolate environment.

Species response to Pleistocene sea-level oscillations and more recent examples of complete marine habitat annihilation (Barber et al. 2002; volcanic eruption on Krakatau) demonstrate the very high recolonization potential of marine species in the IMA. However, the decreasing overall quality of marine habitat can prevent these mechanisms from working properly while increasing the importance of large-scale local and global efforts to reduce marine pollution, control unchecked exploitation, and support an ecologically sensible use and management of marine resources.

## Conflict of Interest

None declared.

## Data Accessibility

DNA sequences have been deposited in GenBank (Tables S1). Microsatellite Data will be deposited in DRYAD.

## Supporting Information

**Table S1.** Genbank accession ID's for CR sequences of *A. perideraion* used in this study. **Table S2.** Population pairwise differences (F_st_, below diagonal) between all *A. perideraion* sampling sites using data from ten microsatellite loci. Bold values denote significance at P ≤ 0.05 (above diagonal) after correction for multiple testing (Benjamini and Hochberg, False Discovery Rate procedure) **Figure S1.** Data from 10 microsatellite loci were used to produce groupings for 290 samples of *A. perideraion* in STRUCTURE (ver. 2.2., Pritchard *et al*.). Depicted here is the probability that each number of groupings applied during the analysis (*k* = 1-10) constitutes the correct subdivision of the dataset (Bayesian likelihood). The length of the burn-in period was set at 120000 and the number of MCMC reps to 300000, with 10 iterations for each *k*. Zero values were entered as 1E-99.
