# Supplementary material for: Limited connectivity and a phylogeographic break characterize populations of the pink anemonefish, Amphiprion perideraion, in the Indo-Malay Archipelago: inferences from a mitochondrial and microsatellite loci
Source: Ecol Evol. 2015 Mar 25;5(8):1717–33. doi: 10.1002/ece3.1455 (PMC4409419; doi:10.1002/ece3.1455)
Supplement: Supplementary file 1 [file ece30005-1717-sd1.doc]

**Table S1** Genbank accession ID’s for CR sequences of *A. perideraion* used in this study.

**Table S2** Population pairwise differences (F_st_, below diagonal) between all *A. perideraion* sampling sites using data from ten microsatellite loci. Bold values denote significance at P ≤ 0.05 (above diagonal) after correction for multiple testing (Benjamini and Hochberg 1995, False Discovery Rate procedure)

**Figure S1** Data from 10 microsatellite loci were used to produce groupings for 290 samples of *A. perideraion* in STRUCTURE (ver. 2.2., Pritchard *et al.* 2000). Depicted here is the probability that each number of groupings applied during the analysis (*k* = 1-10) constitutes the correct subdivision of the dataset (Bayesian likelihood). The length of the burn-in period was set at 120000 and the number of MCMC reps to 300000, with 10 iterations for each *k*. Zero values were entered as 1E-99.
